# Digital Cognitive Behavioural Therapy for Insomnia versus sleep hygiene education: the impact of improved sleep on functional health, quality of life and psychological well-being. Study protocol for a randomised controlled trial

**DOI:** 10.1186/s13063-016-1364-7

**Published:** 2016-05-23

**Authors:** Colin A. Espie, Annemarie I. Luik, John Cape, Christopher L. Drake, A. Niroshan Siriwardena, Jason C. Ong, Christopher Gordon, Sophie Bostock, Peter Hames, Mhairi Nisbet, Bryony Sheaves, Russell G Foster, Daniel Freeman, Joan Costa-Font, Richard Emsley, Simon D. Kyle

**Affiliations:** Sleep and Circadian Neuroscience Institute, Nuffield Department of Clinical Neurosciences, University of Oxford, Sir William Dunn School of Pathology, Oxford, UK; Big Health Ltd., London, UK; Division of Psychology and Language Sciences, Faculty of Brain Sciences, University College London, London, UK; Department of Sleep Disorders and Research Center, Henry Ford Health System, Detroit, MI USA; School of Health and Social Care, University of Lincoln, Lincoln, UK; Rush University Medical Center, Chicago, IL USA; CIRUS Centre for Integrated Research and Understanding of Sleep, Woolcock Institute of Medical Research, University of Sydney, Sydney, Australia; Sleep and Circadian Neuroscience Institute, Department of Psychiatry, University of Oxford, Warneford Hospital, Oxford, UK; The London School of Economics and Political Science, London, UK; Centre for Biostatistics, Institute of Population Health, The University of Manchester, Manchester Academic Health Science Centre, Manchester, UK

**Keywords:** Insomnia, Sleep, CBT, Digital, Health, Function, well-being

## Abstract

**Background:**

Previous research has demonstrated that digital CBT (dCBT), delivered via the Internet, is a scalable and effective intervention for treating insomnia in otherwise healthy adults and leads to significant improvements in primary outcomes relating to sleep. The majority of people with insomnia, however, seek help because of the functional impact and daytime consequences of poor sleep, not because of sleep discontinuity per se. Although some secondary analyses suggest that dCBT may have wider health benefits, no adequately powered study has investigated these as a primary endpoint. This study specifically aims to investigate the impact of dCBT for insomnia upon health and well-being, and will investigate sleep-related changes as mediating factors.

**Methods/design:**

We propose a pragmatic, parallel-group, randomised controlled trial of 1000 community participants meeting criteria for insomnia disorder. In the DIALS trial (Digital Insomnia therapy to Assist your Life as well as your Sleep), participants will be randomised to dCBT delivered using web and/or mobile channels (in addition to treatment as usual (TAU)) or to sleep hygiene education (SHE), comprising a website plus a downloadable booklet (in addition to TAU). Online assessments will take place at 0 (baseline), 4 (mid-treatment), 8 (post-treatment), and 24 (follow-up) weeks. At week 25 all participants allocated to SHE will be offered dCBT, at which point the controlled element of the trial will be complete. Naturalistic follow-up will be invited at weeks 36 and 48. Primary outcomes are functional health and well-being at 8 weeks. Secondary outcomes are mood, fatigue, sleepiness, cognitive function, productivity and social functioning. All main analyses will be carried out at the end of the final controlled follow-up assessments and will be based on the intention-to-treat principle. Further analyses will determine whether observed changes in functional health and well-being are mediated by changes in sleep. The trial is funded by Big Health Ltd.

**Discussion:**

This study will be the first large-scale, specifically designed investigation of the health and well-being benefits of CBT for insomnia, and the first examination of the association between CBT-mediated sleep improvement and health status.

**Trial registration:**

ISRCTN60530898.

**Electronic supplementary material:**

The online version of this article (doi:10.1186/s13063-016-1364-7) contains supplementary material, which is available to authorized users.

## Background

### The importance of insomnia

Insomnia disorder comprises a complaint of poor sleep, with associated significant daytime effects, occurring at least 3 nights per week for at least 3 months [1]. Worldwide, epidemiologic studies report the prevalence of a chronic clinical insomnia disorder at 10 to 12 % [2–4]. Although prevalence is high, natural remission is low. In one study, 74 % of those with insomnia continued to have insomnia a year later and 46 % reported insomnia persisting for over 3 years [5]. Traditionally considered as ‘secondary’, subsumed as symptoms of other clinical diagnoses within mental health care, the recently revised *Diagnostic and Statistical Manual of Mental Disorders, version 5* (DSM-5) outlines the ‘need for independent clinical attention of a sleep disorder’ (p. 1) [1]. This is supported by research demonstrating not only that rates of mental and physical health co-morbidity are high, but that pre-existing chronic insomnia is an independent risk factor for development of depression [6], cardiovascular disease [7] and type 2 diabetes [8, 9]. From the standpoint of public health and well-being, sleep appears to be a more important matter than has been hitherto recognised [10, 11].

### The relationship between poor sleep, daytime functioning and quality of life

Typically, insomnia is associated with increased fatigue, impaired work productivity, reduced quality of life and relationship satisfaction, as well as increased ill-health [12–14]. Despite such evidence of poor functioning being attributed to poor sleep, and also being an essential diagnostic criterion for insomnia, there has been comparatively little research on quality of life. This is all the more surprising given that the perceived impact on personal functioning serves as an important driver of complaint and of help-seeking behavior rather than simply perceived sleep loss [15, 16]. In one large epidemiological study, four of the five most commonly cited reasons for seeking a sleep consultation with a health professional, were daytime consequences of fatigue, psychological distress, physical discomfort, and reduced work productivity [16]. Clinician reports of patient consultations, and cross-sectional and prospective questionnaire studies [17, 18] further demonstrate that individuals with insomnia complain of deficits in mood and cognitive abilities (concentration, memory, attention), coupled with elevated levels of anxiety, fatigue and physical pain/discomfort. Thus, once a threshold of noticeable effect on one’s life is reached, such individuals may feel motivated to seek medical advice.

### Cognitive behavioural therapy (CBT) for insomnia

CBT, regarded as the treatment of first choice for persistent poor sleep [19–21] is a psychological treatment designed to break the patterns of maladaptive thinking and behaviour that serve to maintain insomnia. CBT comprises a range of techniques including a behavioural component (stimulus control, sleep restriction, relaxation) combined with a cognitive (managing sleep-related worries, the racing mind and intrusive thoughts) and an educational (sleep hygiene) component. Meta-analyses indicate that CBT has moderate to large and durable effects on sleep quality, sleep efficiency, sleep onset latency and wake time after sleep onset [22–24]. Moreover, approximately 60 % of those who receive CBT respond to treatment and 39 % achieve remission [25]. What is much less well-established is the effect that CBT may have upon the daytime symptom and functional health profile of people with insomnia. Logically, effective treatment should alleviate such impairments; and furthermore, based on the evidence that impaired sleep may be causally related to reduced quality of life domains (well-being and impaired daytime functional status), improving sleep should improve functioning. There is some preliminary evidence from secondary analyses that CBT may yield generalised benefits [26–29], and even some primary data in small samples that CBT for insomnia may reduce depressive or anxiety symptoms [30, 31], but an adequately powered, definitive trial examining functional health status and well-being is long overdue.

### The current study

This study seeks to ascertain the impact of improved sleep on three key areas of quality of life: functional health status, patient-generated (sleep-related) quality of life impairment and psychological well-being. Over the past 5 years, self-help CBT delivered via the Internet has been introduced, not least because of the importance of widening access to effective psychological therapy. Several randomised controlled trials (RCTs) have evaluated digital CBT (dCBT) applications; each of which has found moderate to large improvements in insomnia symptoms relative to waitlist groups [32–34]. Only one programme, however, has been tested versus a placebo intervention, demonstrating significant and sustained improvements in sleep continuity; for example, sleep diary-assessed sleep efficiency, sleep onset latency and wake time after sleep onset, and self-reported global sleep quality [34]. It is this dCBT intervention that will be used in the present study. Data from the programme show that more than 80 % of participants complete the course within 8 weeks.

The primary hypotheses for the trial are that, compared to sleep hygiene education (SHE):

1. The dCBT intervention will be associated with improved functional health status by the end of treatment (8 weeks)
2. The dCBT intervention will improve positive psychological well-being by the end of treatment (8 weeks)
3. The dCBT intervention will be associated with reduced patient-generated sleep-related quality of life impairment (8 weeks)
4. The effect of dCBT on outcomes (8 weeks) will be mediated by sleep status during the treatment phase (4 weeks)

The secondary hypotheses are that, compared to SHE:

1. The dCBT intervention will be associated with reduced symptoms of negative mood, fatigue and relationship/social dysfunction by the end of treatment (8 weeks)
2. The dCBT intervention will be associated with reduced problems with sleepiness, cognitive impairment and productivity by the end of treatment (8 weeks)
3. Improvements will be maintained at follow-up (24, 36, 48 weeks)
4. The effect of dCBT on longer-term outcomes (24, 36, 48 weeks) will be mediated by sleep status during and upon completion of the treatment phase (4, 8 weeks)

## Methods/design

### Research design

**Fig. 1:**
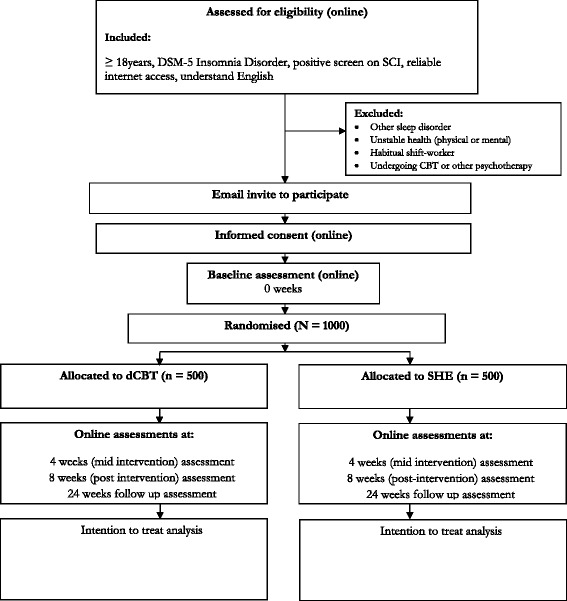
Summary of the trial design for the Digital Insomnia therapy to Assist your Life as well as your Sleep (DIALS) study. *SCI* Sleep Condition Indicator, *dCBT* digital cognitive behavioural therapy, *SHE* sleep hygiene education

The study is a parallel-group, superiority, RCT of dCBT (+ treatment as usual, TAU) versus SHE (+TAU). The trial design is summarised in Fig. 1. The study will be carried out completely online. Participants will access the screening assessment, participant information statement, informed consent form, allocation to condition and intervention, and baseline and follow-up assessments via web or mobile platforms (see also Additional files 1 and 2). Participants will be able to print a copy of the participant information statement and will have the opportunity to contact the researchers via phone or email before consenting. The study has received ethical approval from the University of Oxford Medical Sciences Inter-Divisional Ethics Committee (ref MS-IDREC-C2-2015-024) and is registered at ISCRTN (ISRCTN60530898).

### Participants

We will recruit 1000 community participants. Our inclusion criteria comprise: (1) a positive screen for probable DSM-5 insomnia disorder, (2) a test score of ≤ 16 on the Sleep Condition Indicator (SCI) [35], (3) being aged 18 or older (no upper age limit), (4) having reliable Internet access at home or at work, and (5) being able to read and understand English. We will screen for co-morbid conditions and medication use at baseline but exclude only those people whose health may be considered to be unstable such as significant current symptoms of (a) an additional sleep disorder (e.g. excessively sleepy and possible obstructive sleep apnoea), (b) psychosis or mania, (c) serious physical health concerns necessitating surgery or with a prognosis less than 6 months, (d) those undergoing a psychological treatment programme for insomnia with a health professional, and (e) habitual night shift, evening, or rotating shift-workers. Information covering (a) to (e) will be gathered using online self-report questions, there will be no medical examinations or access to medical records. We will not omit participants who take medication for sleep problems, or for any other physical or mental health problems providing they report their health to be stable. The study will recruit through several channels. These may include online, print and broadcast media announcements or advertisements and the use of contact lists where adults who have volunteered to be involved in research will be re-contacted (See Additional file 3). For example, following completion of open access sleep surveys such as the Great British Sleep Survey (GBSS: www.greatbritishsleepsurvey.com or World Sleep Survey, WSS: www.worldsleepsurvey.com, which generate data on tens of thousands of respondents). Potential participants will also be alerted to the study by information placed on the Sleepio® website (www.sleepio.com) and on the Sleepio® App site (which generate data on hundreds of thousands of respondents). Participants will not receive reimbursement for their participation, but will be provided with access to the dCBT programme free of charge, regardless of allocation to experimental condition. Informed consent will be obtained from all participants.

### Randomisation and allocation concealment

This study will use simple randomisation with an allocation ratio of 1:1, as recommended for large clinical trials [36]. It will be carried out by the automated online system. Hence the research team will be unable to influence randomisation, and will have no access to future allocations.

### Blinding

This is a single-blind trial. Self-report assessments will be completed online and hence the research team will be blind to outcomes during the trial. Participants will be informed of their randomisation outcome by an automatic email, and so they will not be blind to treatment allocation. The research team will have limited contact with research participants and will be unable to bias the allocation or influence the assessments. If participants do contact the team and reveal the allocation, the assessments will remain blinded. Statistical analyses will be conducted by an independent researcher (RE; University of Manchester) who will have access to all data.

### Assessment points

Assessments will take place at weeks 0 (baseline), 4 (mid-treatment), 8 (post-treatment), and 24 (follow-up). In consideration of ethical matters, at week 25 all participants in the control group will be offered dCBT to help with their sleep problems, and so at that point the controlled element of the trial will be complete. Thereafter, there will be a naturalistic follow-up. All participants will be invited to complete further assessments at weeks 36 and 48.

### Planned intervention

Digital cognitive behavioural therapy will be delivered using the Sleepio® programme [34] (www.sleepio.com and associated Sleepio® App). The programme is fully automated and its underlying algorithms feed the delivery of information, support, and advice in a personally tailored manner. Delivery is structured into six sessions, lasting an average of 20 minutes each. Certain tools (such as sleep diaries and relaxation audios) can also be accessed using the web browser of any smartphone. All of the six core sessions, sleep diaries, relaxation audios, and the scheduling tool can also be accessed using an iOS App, but this is only an option for participants who have an iPhone®. The treatment content is based on CBT for insomnia manuals [26–28] and includes a behavioural component (sleep restriction, stimulus control, and relaxation), a cognitive component (paradoxical intention, cognitive restructuring, mindfulness, positive imagery, and putting the day to rest) and an educational component (psycho-education and sleep hygiene).

The programme is highly interactive, and content is presented by an animated virtual therapist. Participants make a time for the session and are prompted via email and/or short text message (SMS) if they do not ‘attend’. Participants complete daily sleep diary information throughout the intervention, which is used by the programme to provide tailored, personalised help. Participants receive an email and/or SMS reminder each morning to prompt them to fill in their sleep diary. In addition, participants complete a short questionnaire at the beginning of therapy to set treatment goals. Throughout the course of therapy, participants have access to a moderated online community and an online library of information about sleep. Participants can view their online case file, which includes four sections: a progress review, a reminder of strategies to try out between sessions, an agreed sleep schedule, and a list of further reading. The system provides online analytics, which can be used to monitor adherence by assessing how many sessions were completed and the number of weeks to complete the course. All information gathered for the programme will be stored in encrypted form on secure servers. Passwords are stored in encrypted form and all sensitive traffic is transmitted securely via SSL by default. Participants will have access to the intervention for up to 12 weeks. Digital CBT will in effect be dCBT + TAU because there will be no requirement for participants to alter their usual care in any way. Physicians, for example, will be free to offer appointments, to prescribe, and to maintain/discontinue prescriptions as they see fit.

Sleep hygiene education (SHE) has been selected for the control arm because this is what people with insomnia are offered most typically in routine care. To ensure consistency of approach and content, SHE will be delivered on a dedicated website where materials can be viewed and downloaded. SHE will be based on recognised sleep hygiene advice [37–39] and will comprise behavioural advice concerning both lifestyle factors and environmental factors associated with sleep and sleeplessness. The latter in particular will focus on creating the optimal bedroom environment for good sleep. The content of SHE will cover the importance of limiting caffeine, nicotine, and alcohol and of carefully managing diet and exercise (lifestyle), as well as limiting noise and light, managing room temperature and body temperature, and improving air quality and bed comfort (environment). SHE will in effect be SHE + TAU because again there will be no requirement for the usual care of participants to be altered in any way.

### Outcome measures

Participants will be prompted by email to complete the assessments in an online, secure environment (www.surveygizmo.eu). The order of the assessments will be consistent across all participants and all time-points. If participants do not complete measures within 2 days they will receive further email reminders. The full battery of questionnaires amounts to around 100 items in total, and takes 20–25 minutes to complete. We have evidence from a recently completed protocol that data on such measures can be successfully collected online [40]. Demographic and descriptive clinical data will be gathered at baseline only. Measurements to permit health economic evaluation will form part of the descriptive demographic data, with some aspects audited at each assessment point (e.g. medication use, visits to health professionals, other health care utilisation). Descriptive data on health behaviours such as smoking and alcohol use will also be reported.

The co-primary measures that relate to functional health and well-being will be the Patient Reported Outcome Measurement Information System: Global Health scale [41] (PROMIS-10), the Warwick-Edinburgh Mental Well-being Scale [42] (WEMWBS) and the Glasgow Sleep Impact Index [43] (GSII). The PROMIS-10 is a reliable (*α* ≥ .92) but brief (10 items), generic measure that has proven to be very useful in measuring outcomes in clinical trials (http://www.nihpromis.org/science/). It can also be used to estimate cost-utility using QALYs (quality-adjusted life-years) [44, 45]. The WEMWBS is a short (14 items) and psychometrically robust measure (*α* ≥. 91) of mental well-being and is included because it focuses entirely on positive aspects of functional mental health. A previous pilot trial suggests the WEMWBS is sensitive to changes after CBT for insomnia [46]. The GSII is a patient-reported outcome (PRO) measure that asks patients to individually generate, and then assess, three domains of sleep-related impairment unique to their own individual context. Its strength is ecological validity and the GSII has been shown to be sensitive to change following CBT [43]. This combination of PRO, generic functional health status, and positive mental state (rather than symptom reduction) matches our intention to evaluate the impact of dCBT upon quality of life domains.

Secondary outcomes relate to specific measurement of the six areas of daytime consequence that are associated with the clinical diagnosis of insomnia disorder [1, 14, 47]. These are mood (Patient Health Questionnaire (PHQ-9: 9 items [48]) and Generalised Anxiety Disorder (GAD-7: 7 items [49])); energy (Flinders Fatigue Scale (FFS: 7 items [50]); relationship satisfaction (Relationship Assessment Scale (RAS: 7 items [51]); cognitive functioning (Cognitive Failures Questionnaire (CFS: 25 items [52])]; work performance and satisfaction (Work Productivity and Activity Impairment questionnaire: Specific Health Problem (WPAI:SHP: 6 items [53]), 1 item on job satisfaction [54]); and sleepiness (Epworth Sleepiness Scale (ESS: 8 items [55]). As an exploratory measure, participants will also complete 1 item about their general life satisfaction [56].

In order to appraise the mediating effects of sleep improvement per se we will use the SCI [35] and estimates of sleep diary parameters [57]. The SCI is an internally consistent (*α* = .86) measure with a clinical cut off that can correctly identify 89 % of those with probable DSM-5 insomnia disorder. The SCI and sleep diary variables have proven to be sensitive to change following dCBT [34].

In addition to these formal assessments the web/mobile platform will provide online analytics for the dCBT group. These can be used, for example, to measure the process of change (sleep diary) and to monitor how many sessions were completed and the number of weeks to complete the course. These will be used in exploratory analyses. We will also gather information on the demographics of the sample, employment, work satisfaction and economic outcomes, their health characteristics, and their use of clinical services during the period of their trial participation.

### Assessment of safety

The likelihood of serious adverse events occurring during this trial is low since dCBT for insomnia has not been reported to cause them. The intervention offered in the trial has previously been tested in a RCT testing change in insomnia and no adverse outcomes were reported [29, 34]. However, studies have shown that daytime sleepiness and vigilance impairment may increase during sleep restriction therapy (SRT) (one component of CBT-I), owing to restricted sleep opportunity [58]. We will record the occurrence of any serious adverse events in trial participants, defined as: (1) all deaths, (2) suicide attempts, (3) serious violent incidents, (4) admissions to secure units, and (5) formal complaints about the online intervention. Owing to the online nature of the assessments and intervention, it is unlikely that the research team will become aware of all such events. At the end of treatment we will also ask participants to complete an adapted version of a previously employed adverse effects measure [59] to assess differential rates of self-reported adverse effects.

### Sample size calculation

Our planned primary intention-to-treat analyses will compare dCBT + TAU versus SHE + TAU for each of the three primary outcomes separately. Assuming a significance level of 1.667 % (adjusted from 5 % because of having three primary outcomes) and a power of 90 %, to detect a standardised effect size of 0.25 we require a minimum of 433 participants in each of the groups in the analysis. Accounting for a conservative dropout rate of 13 %, we will recruit 500 participants in each treatment group, or 1000 participants in all. This sample size will have more than 80 % power to detect a large-sized indirect effect through the sleep mediator (proportion mediated approximately 75 %) for each between-group comparison. We believe that a sample of this size is feasible to recruit for two reasons. First, online/mobile interventions are much more accessible than in person interventions; and second, published research using dCBT has already shown that large samples may be recruited over comparatively short recruitment periods.

### Statistical analysis

All analyses will be carried out using Stata [60]. In accordance with Consolidated Standards of Reporting Trials (CONSORT) guidelines, we will report all participant flow. Descriptive statistics of recruitment, dropout and completeness of interventions will be provided.

The main efficacy analysis will be via intention-to-treat including all participants, with no planned interim analysis for efficacy or futility. Baseline characteristics will be presented by randomised group without formal statistical tests. We will test the primary hypothesis for between-group change in the primary outcomes at 8 weeks using analysis of covariance with baseline outcome measure and treatment assignment as fixed effects, and apply standard regression diagnostics. The analysis will use statistical techniques for handling missing outcome data under a missing-at-random assumption. The secondary outcomes will be analysed using an analogous method, as will subsequent measures of the primary outcomes at 24 weeks. Analysis of all treatment effects will be undertaken after all 24-week outcome measures are completed.

We will use modern causal inference methods to investigate the mediation hypothesis [61]. If the efficacy analysis shows significant between-group differences in the SCI at 4 and 8 weeks, then we will use parametric regression models to test for the indirect effect of SCI on outcomes, and the residual direct effect of treatment on outcomes at 8 and 24 weeks respectively. Since all the measures are continuous, the indirect effects are calculated by multiplying relevant pathways and bootstrapping is used to produce valid standard errors for the indirect effects. All analyses will adjust for baseline measures of the SCI, outcomes and putative measured confounders. Mediation analyses are potentially biased by measurement error in mediators and hidden confounding between mediators and outcomes and we will investigate the sensitivity of the estimates to these problems.

### Dissemination policy

Results from this study will be published in peer-reviewed scientific papers, and presented at major national and international clinical scientific meetings. Findings, wherever possible, will be made accessible online, dependent upon journal policies.

## Discussion

It is already well-established that CBT is the treatment of first choice for people with chronic insomnia, and that sleep-related outcomes, whether on index measures of insomnia or on derivations from sleep diaries, show sustained improvement [19–21]. A recent definitive placebo-controlled RCT has also demonstrated that dCBT yields effect sizes that mirror conventionally delivered face-to-face therapy [34]. What is yet to be established is whether or not CBT for insomnia is causally associated with changes in functional health, quality of life and psychological well-being. This is crucial for two reasons. First, a diagnosis of insomnia disorder cannot be made unless there are clear attributed daytime consequences of night-time poor sleep; and second, it is the degradation of people’s lived experience and quality of life that often leads to clinical complaint and help-seeking behaviour. An investigation of such as primary outcomes is long overdue. This study will be the first specifically designed investigation of the health and well-being benefits of CBT for insomnia, and the first large-scale test of the relationship between CBT-mediated sleep improvement and health status. The results can be expected to influence care provision for the 10–12 % of the adult population who have persistent insomnia problems. In particular, the results may indicate that sleep is a novel therapeutic target in relation to generalised health benefit and improved quality of life. Moreover, because we will be using a dCBT approach, a scalable solution to insomnia may be demonstrated as both viable and effective.

## Trial status

Recruitment will begin in December 2015. It is anticipated that recruitment will be complete in Summer 2016. Therefore, trial results will become available in late 2016.

## Abbreviations

CBT, cognitive behavioural therapy; CFS, Cognitive Failures Questionnaire; CI, Chief Investigator; dCBT, digital cognitive behavioural therapy; DSM-5, Diagnostic and Statistical Manual of Mental Disorders, 5th edition; ESS, Epworth Sleepiness Scale; FFS, Flinders Fatigue Scale; GAD-7, Generalised Anxiety Disorder assessment (7-item version); GBSS, Great British Sleep Survey; GSII, Glasgow Sleep Impairment Index; PHQ-9, Patient Health Questionnaire – 9- item version; PROMIS-10, Patient Reported Outcomes Measurement Information System, Global Health scale (10 items); QALY, quality-adjusted life-years; RAS, Relationship Assessment Scale; RCT, randomised controlled trial; SCI, Sleep Condition Indicator; SCNi, Sleep and Circadian Neuroscience Institute; SHE, sleep hygiene education; TAU, treatment as usual; WEMWBS, Warwick- Edinburgh Mental Well-being Scale; WPAI,SHP, Work Productivity and Activity Impairment questionnaire, Specific Health Problem; WSS, World Sleep Survey

## Additional files

Additional file 1: Participant Information Sheet. (DOC 63 kb) Additional file 2: Consent Page. (DOC 61 kb) Additional file 3: Recruitment text. (DOC 24 kb)
