# Supplementary material for: Digital Cognitive Behavioural Therapy for Insomnia versus sleep hygiene education: the impact of improved sleep on functional health, quality of life and psychological well-being. Study protocol for a randomised controlled trial
Source: Trials. 2016 May 23;17:257. doi: 10.1186/s13063-016-1364-7 (PMC4877942; doi:10.1186/s13063-016-1364-7)
Supplement: Additional file 1: — Participant Information Sheet. (DOC 63 kb) [file 13063_2016_1364_MOESM1_ESM.doc]

**Additional file 1**


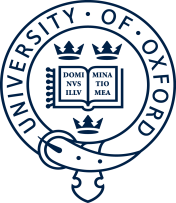

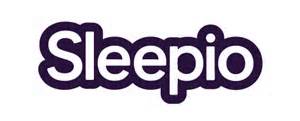


**Participant Information Sheet**

**Digital Insomnia therapy to Assist your Life as well as your Sleep (DIALS) Study**

We would like to invite you to take part in a research study. This webpage should provide you with all the information you need to decide whether or not you’d like to take part. If we’ve missed anything, do get in touch with the [team](#here) and we’ll be happy to answer your questions.

**Key information:**

• This study is for people with current sleep problems who are aged 18 and above and who have reliable access to the internet.

• The aim is to find out whether digital Cognitive Behavioural Therapy (dCBT) for Insomnia can improve health, quality of life and well-being and whether any changes are the result of changes in sleep.

• Everyone who takes part will be given access at no cost to a digital sleep improvement programme delivered via web and mobile (Sleepio [www.sleepio.com](http://www.sleepio.com/)). The programme consists of 6 weekly sessions which take about 20 minutes each to complete. Depending on which group you are assigned to, access will be given either directly or after 6 months.

• Participants will be assigned at random into one of two groups:

- Group A will be offered *Sleep programme 1* (dCBT for insomnia).
- Group B will be offered *Sleep programme 2* (sleep hygiene education). They can take *Sleep programme 1* after 6 months if they so wish.

• If you take part you will therefore have a 50% chance of getting access to *Sleep programme 1* now and 50% chance of getting access in 6 months.

• We’ll ask everyone to fill in online questionnaires to investigate changes in sleep and well-being at the following time points: at the start of the study, after 4 weeks, 8 weeks, 24 weeks, 36 weeks and 48 weeks.

**What is the purpose of this study?**

We want to find out if digital Cognitive Behavioural Therapy can improve health and well-being and whether any changes are the result of changes in sleep. In particular we are interested in the impact that sleep has on quality of life, psychological well-being, mood, energy, relationships, concentration, productivity and sleepiness.

To find out whether better sleep improves people’s health, quality of life and well-being, we are offering participants an online / mobile phone delivered course, proven (through previous research) to improve sleep. We want to see whether those people who receive this course immediately see any changes in their health, quality of life and well-being in comparison to those people who receive sleep hygiene education.

**Why is the study important?**

Adults experience problems with their sleep on a regular basis. Not only do people find it difficult to sleep, they also experience lack of energy, upset mood and poor concentration. It is often such daytime effects on health, wellbeing and quality of life that lead people to seek help.

**Why have I been invited to take part?**

You have been invited to take part because you may have a sleep problem, and may be interested in an insomnia research study. It could be that you have seen a notice, news story or advertisement about our research, or have expressed an interest in volunteering for future research projects, following completion of open access sleep surveys such as the Great British Sleep Survey (GBSS: www.greatbritishsleepsurvey.com or World Sleep Survey (WSS: [www.worldsleepsurvey.com](http://www.worldsleepsurvey.com/)). You might also have expressed your interest in taking part via the Sleepio website (www.sleepio.com) or on the Sleepio App site. We are looking for around 1000 people to take part. Participants must be aged 18 or older, have access to the internet at work or at home and be able to read and understand English.

**Do I have to take part?**

No, it is up to you to decide whether to take part or not.

**What will happen to me if I take part?**

Everybody who takes part will complete an online assessment at the beginning and then after 4 weeks, 8 weeks, 24 weeks, 36 weeks and 48 weeks. The online assessment is made up of a series of questionnaires asking about your sleep, and various aspects of your health and wellbeing. These are all questionnaires that have been used in other studies. You can complete the assessment wherever is most convenient for you, as long as you have access to the internet.

After you complete the first assessment, it will be decided at random if you will receive *Sleep programme 1* or *Sleep programme 2*. Those who are assigned to *Sleep programme 2* will also have access to *Sleep programme 1* after 6 months in the study. The decision about who will receive which sleep programme immediately is made by an automated computer system (rather like throwing a dice). Everyone gets a 50% chance of being assigned to either sleep programme. The reason we need people to start with different sleep programmes is so that we can compare the effects of the two programmes. It is only by doing this that we can be sure that any changes are due to a specific programme we are offering.

You will be prompted to complete any questionnaires and to enter the website for the sleep programmes by a series of automated emails. This is the only way we will contact you, and there will be no face-to-face contact with the research team. If however you have any difficulties throughout your time in the study you are very welcome to make contact with the research team using the contact details provided below.

When you come to access *Sleep programme 1* you will be sent a code to enter a separate website that provides the programme. You will then need to answer some extra questions about yourself and your sleep. Some of these questions will be the same as the ones you complete as part of the initial assessment. We apologise for the repetition; the sleep programme website is separate from the study website and will need your information to provide help that is personal to you.

In total you will receive six online sessions in *Sleep programme 1,* all focused on relieving your sleep problems. This will include thinking about things that you can do differently both in the day and at night, adjusting unhelpful thinking patterns that get in the way of you sleeping and calming negative emotions. As well as the weekly sessions you will also have access to an online community of other people who have been through the course and many useful fact sheets about various aspects of sleep. Throughout the course you will need to complete a daily online sleep diary so that the website can monitor any changes that happen and adjust the advice you receive accordingly. The website can also help you to set up prompts to remind you to fill these in.

When you come to access *Sleep programme 2*, you will receive an automatic email that will give you access to the dedicated webpages.

**Will I be compensated for my time?**

There shall be no financial or other rewards for participants.

**What are the possible disadvantages, risks or side effects of any examination received when taking part?**

We do not anticipate that there are any risks in taking part.

**Will my taking part in the study be kept confidential?**

The information you provide to the DIALS research team, during the course of the study, will be kept confidential, subject to normal legal requirements. All research data will be anonymised so no-one can be identified. We will not share any individual data with anyone outside of the immediate team. The only exception to this is that responsible members of University of Oxford staff may require access to the data for monitoring and/or audit of the study, to make sure we are complying with all regulations. Maintenance of confidentiality of information is subject to normal legal requirements. You should note that the sleep improvement programme is a separate company with its own terms and conditions regarding how they use the data you provide.

The company is committed to protecting the confidentiality of personal information in any form, complying with best practice in relation to obtaining, recording, holding, using and disclosing information and conforming to statute law (including the Data Protection Act 1998 and the Human Rights Act 1998). Once we send you the link to the sleep improvement programme, you are encouraged to read the ‘terms and conditions’ and ‘privacy policy’ pages before registering for the programme. Lastly, whilst the sleep improvement programme is separate to the DIALS research team, all data that you provide to the programme may be shared with the DIALS research team, but not the other way around.

**What will happen if I don’t want to carry on with the study?**

Participants can withdraw from the study at any time. You do not have to give a reason and your medical care and legal rights will not be affected. Simply contact the researchers using the contact details provided below.

**What if there is a problem?**

The University has arrangements in place to provide for harm arising from participation in the study for which the University is the Research Sponsor. If you were to be upset about anything concerning the research then you would be welcome to speak to Professor Colin Espie (contact details below) who is an experienced clinician.

**What if I have a complaint?**

If you have a concern about any aspect of this project, please speak to Professor Colin Espie (contact details at the foot of this document) who will do his best to answer your query. A researcher will acknowledge your concern within 10 working days and give you an indication of how he intends to deal with it. If you remain unhappy or wish to make a formal complaint, please contact the chair of the Research Ethics Committee at the University of Oxford (Chair, Medical Sciences Inter-Divisional Research Ethics Committee; Email: ethics@medsci.ox.ac.uk; Address: Research Services, University of Oxford, Wellington Square, Oxford OX1 2JD). The chair will seek to resolve the matter in a reasonably expeditious manner.

**What will happen to the results of the study?**

A copy of the results will be made available on the Sleep and Circadian Neuroscience Institute of the Oxford University webpage. You will be emailed a link to this if you have indicated that you are interested in reading the results of the study. The results will also be published in academic journals and discussed at relevant conferences. No person will be identified in the results – we are interested in changes across the two study groups of people, not individuals.

**Who is funding the study?**

The study is funded by Big Health Ltd, the company who have developed the digital insomnia therapy.

**Who has reviewed the study?**

This study has been reviewed by, and received ethics clearance through, the University of Oxford Central University Research Ethics Committee.

**Who is running the study?**

The chief investigator for the study is Professor Colin Espie (Sleep & Circadian Neuroscience Institute, Nuffield Department of Clinical Neurosciences, University of Oxford). The wider study team comprises both members of the University of Oxford and a commercial company which delivers the sleep improvement program.

**CONTACT DETAILS FOR THE TEAM:** You can contact the research team using the following details:

Dr. Annemarie Luik

Email address: annemarie.luik@ndcn.ox.ac.uk, telephone number: +44 (0)1865 618665, postal address: Dr. Annemarie Luik, Sleep & Circadian Neuroscience Institute, Nuffield Department of Clinical Neurosciences, University of Oxford, Sir William Dunn School of Pathology, South Parks Road, OX1 3RE, UK.

Prof. dr. Colin Espie

Email address: colin.espie@ndcn.ox.ac.uk, postal address: Prof. dr. Colin Espie, Sleep & Circadian Neuroscience Institute, Nuffield Department of Clinical Neurosciences, University of Oxford, Sir William Dunn School of Pathology, South Parks Road, OX1 3RE, UK.
