# Supplementary material for: Digital Cognitive Behavioural Therapy for Insomnia versus sleep hygiene education: the impact of improved sleep on functional health, quality of life and psychological well-being. Study protocol for a randomised controlled trial
Source: Trials. 2016 May 23;17:257. doi: 10.1186/s13063-016-1364-7 (PMC4877942; doi:10.1186/s13063-016-1364-7)
Supplement: Additional file 2: — Consent Page. (DOC 61 kb) [file 13063_2016_1364_MOESM2_ESM.doc]

**Additional file 2**


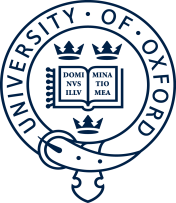

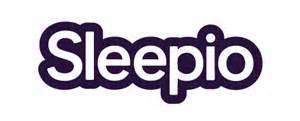


**Consent Page**

**Digital Insomnia therapy to Assist your Life as well as your Sleep (DIALS) Study**

This study is designed to investigate the impact of digital Cognitive Behavioural Therapy for insomnia upon health and wellbeing, and will examine whether any observed changes are the result of changes in sleep.

It is necessary to consent to each of the following statements in order to take part. If any of the following statements are unclear please refer to the Participant Information [instructions for access / hyperlink included here].

I confirm that I have read and understand the Information Page for the DIALS study.

Only if you completed the Great British Sleep Survey: I am happy for any data that I previously provided for the Great British Sleep Survey to be used for the DIALS study.

1. I understand that if I have questions I can contact the study team. If I have asked questions, I confirm that I have received satisfactory answers.
2. I understand that I can withdraw from the study at any point, without penalty, by advising the researcher of my decision.

I understand that the study has received ethical approval by the University of Oxford Central University Research Ethics Committee.

1. I understand who will have access to my personal data, how it will be stored and what will happen to the data after the end of the study.

I consent to information collected as part of the sleep improvement programme being shared with the DIALS research team.

I understand how to raise a concern and make a complaint.

1. I agree to participate in the above study.

Click next

For further information or questions please contact:

Dr Annemarie Luik

Sleep & Circadian Neuroscience Institute, Nuffield Department of Clinical Neurosciences

University of Oxford

Sir William Dunn School of Pathology

South Parks Road

Oxford, OX1 3RE, UK

Email: [annemarie.luik@ndcn.ox.ac.uk](mailto:colin.espie@ndcn.ox.ac.uk)

Tel: +44 (0)1865 618665
