# Supplementary material for: Digital Cognitive Behavioural Therapy for Insomnia versus sleep hygiene education: the impact of improved sleep on functional health, quality of life and psychological well-being. Study protocol for a randomised controlled trial
Source: Trials. 2016 May 23;17:257. doi: 10.1186/s13063-016-1364-7 (PMC4877942; doi:10.1186/s13063-016-1364-7)
Supplement: Additional file 3: — Recruitment text. (DOC 24 kb) [file 13063_2016_1364_MOESM3_ESM.doc]

**Additional file 3**

**Problems sleeping – need some help?**

The University of Oxford, in collaboration with Big Health Ltd, is conducting a study on insomnia.

Most people with insomnia have not only poor sleep, but also problematic daytime effects after a bad night. Therefore, the aim of the study is to find out whether digital Cognitive Behavioural Therapy (that is by web and mobile) can improve health, quality of life and wellbeing as well as poor sleep.

The study is suitable for adults aged 18 years and above who have persistent problems getting to sleep and/ or staying asleep.

For further information about the study and whether or not this might be suitable for you please contact:

Dr Annemarie Luik

Email address: annemarie.luik@ndcn.ox.ac.uk, telephone number: +44 (0)1865 618665, postal address: Dr Annemarie Luik, Sleep & Circadian Neuroscience Institute, Nuffield Department of Clinical Neurosciences, University of Oxford, Sir William Dunn School of Pathology, South Parks Road, OX1 3RE, UK.
